# Functional Limitations in Patients with Diabetic Neuropathy: Prevalence, Optimal Cut-off Points, and Impact on Physical and Mental Health

**DOI:** 10.21315/mjms-09-2025-614

**Published:** 2025-12-31

**Authors:** Narongsak Khamnon, Pairaya Sitthiracha, Phannarin Suwannarat, Areeya Jiamjunyasiri, Chonticha Kaewjoho

**Affiliations:** 1 Department of Physical Therapy, School of Integrative Medicine, Mae Fah Luang University, Chiang Rai, Thailand; 2 Research Group on Smart Integrative Medicine and Technology Sustainability, Mae Fah Luang University, Chiang Rai, Thailand; 3 Department of Anatomy, Faculty of Medicine, Khon Kaen University, Khon Kaen, Thailand; 4 Department of Physical Therapy, School of Allied Health Sciences, University of Phayao, Phayao, Thailand

**Keywords:** diabetes mellitus, peripheral neuropathy, functional limitation, fall, cut-off score

## Abstract

**Background:**

This study aimed to examine the prevalence of functional limitations, compare physical ability and mental health between diabetic participants with diabetic peripheral neuropathy (DPN) who had and did not have functional limitations, and determine the correlations, cut-off points, area under the curve (AUC), sensitivity, and specificity of various physical performance tests for identifying individuals with these limitations.

**Methods:**

The participants’ baseline characteristics and neuropathy severity were assessed. Functional limitations and mental health were evaluated using the 9-Question Depression Screening tool (9Q) questionnaire, while physical abilities were measured by handgrip strength (HG), the Five Times Sit To Stand Test (FTSTS), Timed Up and Go (TUG), the 10-m walk test (10MWT), and the 2-min step test (2MST).

**Results:**

Among the participants with DPN, 58% had at least one functional limitation, most commonly walking up 10 steps (86.8%), performing leisure activities (63.2%), and stooping or rising from a chair (27.9%). Those with limitations had significantly lower mental health, muscle strength, balance, and endurance (*P* < 0.05). Functional limitations correlated moderately with HG, FTSTS, TUG, 2MST, and 10MWT (*P* < 0.001) and mildly with 9Q score. 10MWT showed the strongest predictive ability (AUC = 0.927), while other tests demonstrated moderate to high accuracy (AUC = 0.692–0.899).

**Conclusion:**

Over half of the individuals with DPN experienced at least one functional limitation, most commonly affecting mobility and daily activities. Those with limitations exhibited poorer dynamic balance, muscle strength, and endurance. Among the physical performance tests, 10MWT demonstrated the strongest predictive ability (cut-off = 0.75 m/s, AUC = 0.927), underscoring its reliability and efficiency in identifying functional limitations in this population.

## Introduction

Diabetes is a common and chronic disease that affects millions of people worldwide. According to the 10th edition of the *IDF Diabetes Atlas*, 537 million people live with diabetes globally, 90% of whom have been diagnosed with type 2 diabetes. This number is projected to rise to 643 million by 2030 and to 784 million by 2045. In Thailand, the prevalence of diabetes has steadily increased from 19.07% in 2018 to 20.64% in 2021 (1). Chronic hyperglycemia can damage and impair the function of vital organs, leading to serious complications, such as cardiovascular disease, diabetic retinopathy, nephropathy, and neuropathy (2, 3). Diabetic neuropathy affects between 30% and 50% of individuals with diabetes and is often associated with reduced mobility, an increased risk of falls, and a decline in overall quality of life (4).

Diabetic peripheral neuropathy (DPN), which results particularly from nerve damage in the hands and feet, leads to decreased proprioception and numbness of the lower extremities, which can impair balance and increase the risk of falls and musculoskeletal injuries (5, 6). Beyond these physical complications, recent surveys by the International Diabetes Federation (IDF) have highlighted the psychological burden faced by individuals with diabetes. The ongoing need for lifestyle modification and strict glycemic control can negatively affect mental health, leading to irritability, anxiety, feelings of rejection, and depression (7, 8). Consequently, DPN impacts both physical and mental well-being, thus contributing to a cycle of reduced mobility, increased injury risk, greater functional limitations, and overall poorer quality of life (7–9).

A functional limitation refers to a restriction or inability to perform an action or activity in the manner or within the range considered normal for a human being. It may result from physical, cognitive, or psychological impairments and can affect an individual’s ability to engage in daily life, work, or social activities (10). Previous research has shown that physical abilities differ between individuals with DPN who experience functional limitations and those who do not (11). Functional limitations also occur more frequently in individuals with DPN, and dynamic balance stability has been reported to decline more in these individuals than in non-DPN individuals (12). Despite the clinical importance of these findings, there remains a lack of evidence regarding the prevalence of functional limitations among individuals with DPN. Furthermore, to date, no study has identified specific cut-off points in physical ability test results that distinguish participants with and without functional limitations in daily life, nor has the correlation between the number of functional limitations and overall functional ability been examined. Identifying these thresholds and relationships could assist healthcare providers and community health teams in screening, assessing, and monitoring at-risk patients.

Therefore, this study aims to investigate the prevalence of functional limitations and compare the physical ability and mental health of diabetic participants with peripheral neuropathy who do and do not experience such limitations. It also aims to determine the correlations with functional ability as well as the cut-off points in physical ability test results that may help differentiate between these two groups.

## Methods

### Study Design and Setting

This cross-sectional study was conducted among diabetic patients with peripheral neuropathy living in various communities in northern Thailand. The research protocol was approved by the Institutional Ethics Committee for Human Research (EC 24001-25). Prior to participation, all individuals provided written informed consent. To ensure participant confidentiality, each record was assigned a unique alphanumeric code. The study was carried out in accordance with the principles outlined in the Declaration of Helsinki.

### Participants

Eligible participants were type 2 diabetes patients aged 60 to 79 years with peripheral neuropathy, identified by a Michigan Neuropathy Screening Instrument (MNSI) score of at least 2.5, and able to communicate and follow instructions. The MNSI includes a 15-item questionnaire and a lower extremity physical exam (touch, vibration, and ankle reflex). DPN was defined as abnormal sensory/nerve conduction, at least seven positive questionnaire responses, or an MNSI exam score greater than 2.5 (13). Participants with neurological disorders (e.g., stroke or Parkinson’s disease) that affected mobility and individuals with deformities or abnormalities in their limbs that could interfere with the study were excluded (9). In addition, participants experiencing joint and muscle inflammation or musculoskeletal conditions with a pain score greater than 5 out of 10 on the Visual Analogue Scale that could affect the study were also excluded.

### Sample Size

The number of participants in this study was determined through estimated sample size calculations based on the prevalence of functional limitations (78%) reported by Huang et al. (14), using a sensitivity of 0.65 and a specificity of 0.13. Using a Z_(α/2) value of 1.96 and a margin of error (e) of 0.05, the required sample size was calculated to be no fewer than 140 participants (14).

### Research Procedure

Eligible participants were interviewed and assessed in terms of their demographics (i.e., body weight, height, sex, age, and underlying disease, if any), history of falls, and the consequences of falls within the previous six months. Subsequently, all participants were assessed for functional limitations using items from the National Health and Nutrition Examination Survey (NHANES) questionnaire covering 19 activities from five domains, including daily activities, general physical activities, instrumental activities of daily living, leisure and social activities, and lower extremity mobility. Participants received a score of 1 if they had trouble performing an activity and a score of 0 if they could perform it without difficulty. A cumulative score of 1 or higher indicated that the participant had functional limitations, while a score of 0 indicated no functional limitations (10).

Participants from both groups underwent evaluations of mental and physical health. Mental health status was assessed using the 9-Question Depression Screening tool (9Q), while physical abilities were evaluated through several tests: the Timed Up and Go (TUG) test for dynamic balance, the Five Times Sit To Stand Test (FTSTS) for lower limb strength, the handgrip strength (HG) test for upper limb strength, the 10-m walk test (10MWT) for walking speed, and the 2-min step test (2MST) for endurance in physical activities.

### Measurements

#### NHANES Questionnaire

The NHANES questionnaire assesses functional limitations by asking participants about their ability to perform 19 physical activities, which are grouped into five domains. These include activities of daily living, such as getting in and out of bed; instrumental activities of daily living, which are more complex tasks, such as managing money; leisure and social activities, such as going to movies; lower extremity mobility, which involves walking short distances or climbing stairs; and general physical activities, such as lifting or carrying objects. For each activity, participants report their level of difficulty; a score of 1 is assigned for any degree of difficulty, while a score of 0 is given if there is no difficulty with any activity (10).

#### TUG

Participants were timed on their ability to stand up from an armchair, walk for 3 m, turn around, and walk back to sit down in the chair in the fastest and safest manner. The average time over the three trials was recorded (15).

#### HG

From a sitting position, the participants placed the tested forearm on the armrest of the chair. They then squeezed an HG dynamometer as hard as possible for three trials per hand. The maximal grip strength of both hands was recorded in kilograms (16).

#### FTSST

The participants were timed on their ability to complete five chair-rise cycles in the fastest and safest manner from a chair with an armrest. The average results of the three trials were used for data analysis (16).

#### 10MWT

The 10MWT is a quick, easy, and reliable measure to record walking speed. The participants were timed on their ability to walk at the fastest speed they felt comfortable along the middle 4 m section of a 10 m walkway. The average time over the three trials was converted to a walking speed using the formula walking speed = distance/time (m/s) (16).

#### 2MST

In the 2MST, a cardiopulmonary endurance test, the participants were instructed to perform as many high-knee lifts in place as possible within 2 min. The researcher used a manual counter to record the number of repetitions, counting one complete cycle when both the left and right legs had been lifted (17).

### Statistical Analysis

Descriptive statistics were used to summarise the participant demographics (age, sex, weight, height, and body mass index [BMI]) and the prevalence of functional limitations. The Shapiro–Wilk test was applied to assess data normality. Independent *t*-tests compared group means, while chi-square tests compared proportions. Pearson’s correlation coefficient was used to examine the relationship between the number of functional limitations and the results of the functional ability tests. Inferential statistics, including receiver operating characteristic (ROC) curve analysis, were employed to evaluate differences in physical performance and mental health between participants with and without functional limitations. ROC curve analysis was also used to determine the optimal cut-off values for physical performance tests in identifying functional limitations. Sensitivity was plotted against 1-specificity for all possible cut-off scores, and the area under the curve (AUC) was used to quantify overall test accuracy (range: 0 to 1, with higher values indicating better discrimination) (18). Statistical significance was set at a *P*-value less than 0.05.

## Results

### Participants

A total of 217 individuals who expressed interest in participation were initially enrolled, of whom 148 met the inclusion criteria. These 148 participants, all with DPN, were categorised into two groups: 66 with physical limitations and 82 without. Most of the participants were female and classified as early older adults. When comparing the baseline characteristics between the two groups, there were no statistically significant differences noted but then going on to list such differences for sex, questionnaire, and examination component of the MNSI (*P* < 0.05), as presented in Table 1.

### Prevalence of Functional Limitations among DPN Participants

**Figure 1:**
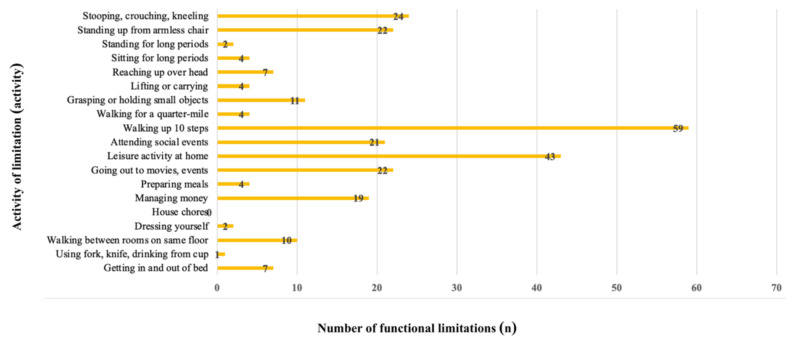
Number of functional limitations

In this study, 148 participants were assessed for functional limitations using NHANES questionnaire items, of whom 86 (58%) had at least one. Participants most frequently reported the following activities as difficult, burdensome, or requiring assistance: walking up 10 steps (*n* = 59, 86.76%); engaging in leisure activities at home, such as gardening or cleaning (*n* = 43, 63.23%); and stooping, crouching, or kneeling and standing up from an armless chair (*n* = 24, 27.91%). These findings are shown in Figure 1.

### Physical Ability among DPN Participants with and without Functional Limitations

Significant differences in mental health status, as assessed by the 9Q questionnaire, were identified between diabetic participants with peripheral neuropathy who had functional limitations and those who did not. Those without functional limitations also performed significantly better (*P* < 0.05) in physical function tests, including TUG, HG, FTSTS, 2MST, and 10MWT, as shown in Table 2.

### Correlation Between the Number of Functional Limitations and Physical Ability among DPN Participants

This study found a significant moderate correlation between the number of functional limitations and HG, FTSTS, TUG, 2MST, and 10MWT (*P* < 0.001). Additionally, a significant mild correlation was observed between the number of functional limitations and the 9Q score, as shown in Table 3.

### Cut-off Values of Physical Ability Tests to Identify Functional Limitations among DPN Participants

**Figure 2:**
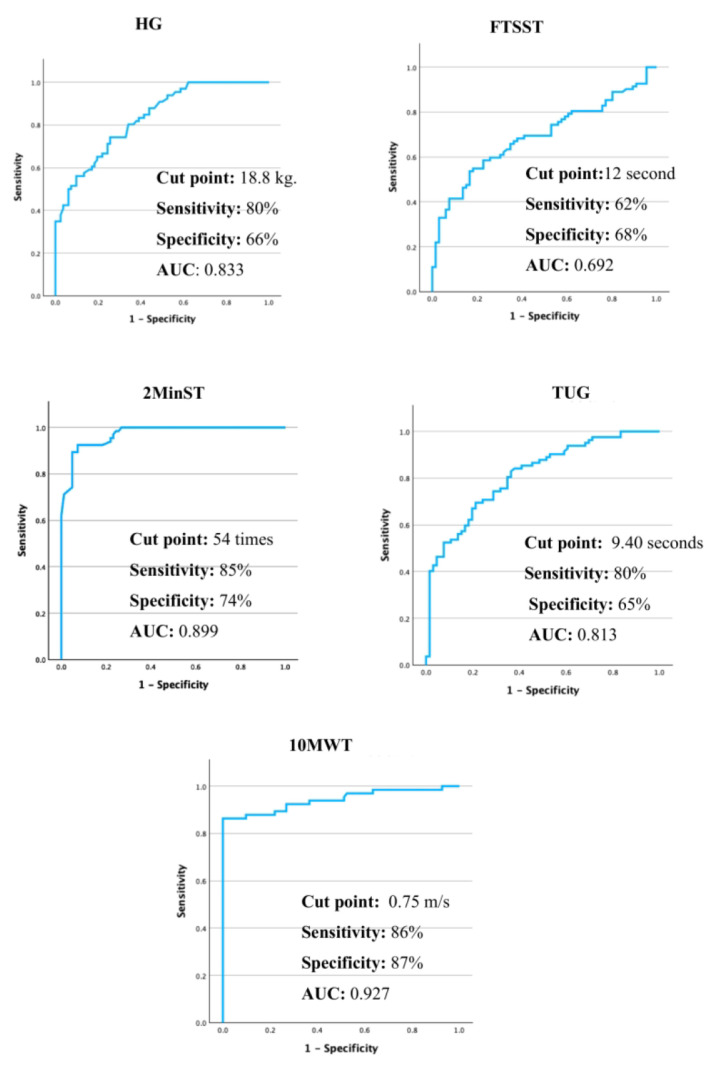
Cut-off values, sensitivity, and specificity of the physical ability test in participants with physical limitations

When determining the optimal physical performance test cut-off scores for identifying functional limitations in diabetic patients with peripheral neuropathy, 10MWT demonstrated the highest predictive accuracy (0.75 m/s; sensitivity = 86%, specificity = 87%, AUC = 0.927). This finding indicates that this test is the most effective tool for detecting at least one functional limitation in this population. The next best performances were observed for 2MST (54 steps; sensitivity = 85%, specificity = 74%, AUC = 0.899), HG (18.8 kg; 80%, 66%, AUC = 0.833), TUG (9.40 s; 80%, 65%, AUC = 0.813), and FTSTS (12 s; 62%, 68%, AUC = 0.692). These results are illustrated in Figure 2.

## Discussion

This study aimed to investigate the prevalence of functional limitations, compare physical ability and mental health in diabetic participants with DPN with and without functional limitations, and identify correlations and cut-off scores for physical ability tests. The results indicated that 58% of the participants with DPN had at least one functional limitation, with the most frequently reported difficulties being walking up 10 steps (86.8%), engaging in leisure activities at home (63.2%), and stooping, crouching, kneeling, and standing up from an armless chair (27.9%). Participants with functional limitations showed significantly lower scores in mental health, muscle strength, balance, and endurance than those without limitations (*P* < 0.05). The number of functional limitations was moderately correlated with HG, FTSTS, TUG, 2MST, and 10MWT (*P* < 0.001) and mildly correlated with the 9Q score. Among the physical ability tests, 10MWT demonstrated the highest predictive ability for identifying at least one functional limitation, with an excellent AUC of 0.927. The other tests (2MST, TUG, HG, and FTSTS) also showed moderate to high accuracy, with AUC values ranging from 0.692 to 0.899.

Among the participants with DPN, over half (58%) reported experiencing at least one functional limitation. The most common difficulties they faced were walking up 10 steps (86.8%), participating in leisure activities at home (63.2%), and performing activities such as stooping, crouching, kneeling, and standing up from an armless chair (27.9%). These results indicate that DPN has a significant impact on increasing the risk of functional limitations among the diabetic population. The findings also showed that individuals with functional limitations exhibited significantly poorer mental health and reduced muscle strength in both upper and lower limbs, balance, and endurance compared to those without such limitations. When considering the characteristics of both groups, those with functional limitations had significantly higher MNSI scores, suggesting a greater likelihood of nerve function impairment. This neuropathic degeneration, especially in the hands and feet, can lead to muscle weakness and sensory loss, impairing balance and gait (19, 20).

Previous research has established a significant relationship between depressive symptoms and glycemic control, aligning with our finding that diabetic patients with functional limitations experience poorer mental health, particularly depression. Reduced physical capacity may further contribute to decreased activity levels or sedentary behaviour, adversely affecting balance (21, 22). Patients with long-standing peripheral neuropathy exhibit greater physical impairment and a lower quality of life than those without neuropathy (23), consistent with our results. In this study, the number of functional limitations was moderately correlated with HG, FTSTS, TUG, 2MST, and 10MWT (*P* < 0.001) and mildly correlated with the 9Q score, whereas Techo et al. (11) reported significantly lower performance scores among diabetic patients with functional limitations. These findings underscore the importance of promoting both psychological and physical functions to improve physical performance and participation among individuals with DPN.

This study also found that a 10MWT walking speed of 0.75 m/s demonstrated the strongest ability to predict functional limitations, with an excellent AUC of 0.927, indicating that 10MWT is the most effective tool for identifying individuals with at least one limitation. Other tests, including 2MST, TUG, HG, and FTSTS, showed moderate to high predictive power, with AUCs ranging from 0.692 to 0.899. 10MWT is a widely used performance measure that assesses walking speed over a short distance, providing valuable insights into functional mobility, gait, and vestibular function. It is strongly correlated with overall health and independence in older adults (24). Li et al. (25) reported that 10MWT effectively distinguished fall risk among older adults with knee osteoarthritis, while Amatachaya et al. (26) found high correlations between 10-m and 6-m walk test speeds, supporting MWT’s concurrent validity (11, 24, 26). These findings highlight that 10MWT is a reliable and efficient tool for evaluating functional mobility and predicting limitations, particularly in populations at risk of impaired physical function.

However, this study has limitations that may affect its generalisability. The sample was predominantly female, and participants had a long duration of diabetes and were in relatively good physical function, potentially limiting the representativeness of the findings to the broader diabetic population. Future research should include more diverse samples in terms of sex, disease duration, and physical function to improve assessment accuracy and applicability.

## Conclusion

This study found that 58% of individuals with DPN had at least one functional limitation, most commonly difficulty walking upstairs, performing leisure activities, and performing tasks requiring stooping or standing up from a chair. Participants with limitations exhibited significantly poorer mental health and reduced muscle strength, balance, and endurance. Functional limitations were moderately correlated with HG, FTSTS, TUG, 2MST, and 10MWT and mildly correlated with the 9Q score. Among the tests, the 10MWT showed the highest predictive ability (0.75 m/s; AUC = 0.927), outperforming the others and highlighting its reliability for identifying functional limitations. These findings underscore the importance of promoting psychological and physical function to improve mobility, participation, and quality of life among individuals with DPN.

## Figures and Tables

**Table 1:** Baseline characteristics of the participants (*n* = 148)

Variable	Total of participants (*n* = 148)		*P*-value

	Non-functional limitations (*n* = 82)	Functional limitations (*n* = 66)	
Age (year) a	68.26 ± 7.99 (65.51, 71.01)	67.83 ± 6.12 (66.12, 69.53)	0.170

Sex, *n* (%) b			
Male (n = 55)	31 (56.4)	24 (43.6)	0.027*
Female (*n* = 93)	35 (37.6)	58 (62.4)	

Body mass index a	19.00 ± 0.51 (17.95, 20.04)	19.5 ± 0.37 (18.77, 20.29)	0.164

Post-diabetic times (months) a	119.91 ± 15.34 (88.73, 151.09)	133.07 ± 13.66 (105.63, 160.51)	

MNSI score a			
Subjective examination (max score = 13)	2.92 ± 1.48 (2.16, 3.44)	3.88 ± 2.20 (2.94, 4.29)	0.004*
Physical examination (max score = 10)	4.96 ± 1.28 (4.56, 5.28)	6.53 ± 0.95 (6.32, 6.74)	0.003*

Blood pressure (mmHg) a			
Systolic	136.21 ± 19.93 (128.48, 143.94)	133.00 ± 22.54 (124.08, 141.91)	0.770
Diastolic	78.04 ± 18.48 (70.86, 85.20)	73.96 ± 12.00 (69.24, 78.71)	0.172

Heart rate (beats per minute) a	81.25 ± 9.69 (77.49, 85.01)	78.93 ± 9.60 (75.12, 82.72)	

Fasting blood sugar (mg/dL) a	132.32 ± 16.82 (125.81, 138.84)	133.33 ± 35.36 (119.34, 147.31)	0.517

Oxygen saturation (%) a	97.21 ± 0.74 (97.24, 96.93)	97.04 ± 1.01 (96.63, 97.44)	0.053

a Values are presented as mean ± standard deviation (95% CI);

b Values are presented as a number (percent);

* Statistical significance *P* < 0.05

**Table 2:** Comparison of mental health status and physical performance between participants with and without functional limitations among a total of 148 participants

Variable	Total of participants (*n* = 148)		*P*-value

	Non-functional limitations (*n* = 82)	Functional limitations (*n* = 66)	
Mental health			
9Q Score (total score = 27) b	1.91 ± 2.74 (0.97, 2.85)	3.23 ± 3.20 (2.40, 4.18)	0.002*

Physical function			
HG (kg) b	25.33 ± 1.20 (22.92, 27.74)	17.83 ± 0.50 (16.21, 18.23)	0.024*
FTSTS (s) b	11.21 ± 0.3 (10.61, 11.82)	14.09 ± 0.57 (13.09, 15.10)	0.035*
TUG (s) b	9.00 ± 0.27 (8.44, 9.55)	12.98 ± 0.75 (11.49, 14.47)	< 0.001*
2MST (time) b	64.36 ± 1.40 (61.56, 67.16)	41.53 ± 1.58 (38.52, 44.49)	0.026*
10MWT m/s) b	1.04 ± 0.46 (0.96, 1.14)	0.62 ± 0.02 (0.59, 0.65)	< 0.001*

9 Q = 9-Question Depression Screening tool; HG = Hand grip test; FTSTS = Five Times Sit To Stand Test; TUG = Timed Up and Go test; 2MST = 2-Minute Step Test; 10 MWT = 10-Metre Walk Test;

b Values are presented as a number (percent);

* Statistical significance *P* <0.05

**Table 3:** The correlation between the number of functional limitations and the functional ability of individuals with diabetic neuropathy

Variable	Mean ± SD (95% CI)	Number of functional limitations	

		rho	*P*-value
9Q Score c	2.49 ± 0.23 (2.09, 2.98)	0.322**	< 0.001
HG (kg) c	20.84 ± 0.69 (19.55, 22.24)	−0.479**	< 0.001
FTSTS (s) c	12.81 ± 0.32 (10.38, 12.31)	0.450**	< 0.001
TUG (s) c	11.21 ± 0.48 (10.38, 12.31)	0.514**	< 0.001
2MST (time) c	51.72 ± 1.40 (48.92, 54.48)	−0.585**	< 0.001
10MWT (m/s) c	0.81 ± 0.03 (0.76, 0.87)	−0.560**	< 0.001

SD = standard deviation; CI = confidence interval; kg = kilogram; s = second; m/s = metre per second; 9Q = 9-Question Depression Screening tool; HG = Hand Grip test; FTSTS = Five Times Sit To Stand Test; TUG = Timed Up and Go test; 2MST = 2-Minute Step Test; 10 MWT = 10-Metre Walk Test;

** Statistical significance *P* < 0.001;

c Pearson’s correlation coefficient was used to analyse the relationship among continuous variables
